# Prevalence of commonly prescribed medications potentially contributing to urinary symptoms in a cohort of older patients seeking care for incontinence

**DOI:** 10.1186/1471-2318-13-57

**Published:** 2013-06-10

**Authors:** Mandavi Kashyap, Le Mai Tu, Cara Tannenbaum

**Affiliations:** 1 Centre de Recherche, Institut Universitaire de Gériatrie de Montréal, University of Montreal, 4545 Queen Mary Road Suite 4824, Montreal, QC H3W 1W5, Canada; 2 Institut Universitaire de Gériatrie de Sherbrooke, Université de Sherbrooke, Sherbrooke, QC, Canada

**Keywords:** Older adult, Lower urinary tract symptoms, Incontinence, Medication

## Abstract

**Background:**

Several medication classes may contribute to urinary symptoms in older adults. The purpose of this study was to determine the prevalence of use of these medications in a clinical cohort of incontinent patients.

**Methods:**

A cross-sectional study was conducted among 390 new patients aged 60 years and older seeking care for incontinence in specialized outpatient geriatric incontinence clinics in Quebec, Canada. The use of oral estrogens, alpha-blocking agents, benzodiazepines, antidepressants, antipsychotics, ACE inhibitors, loop diuretics, NSAIDs, narcotics and calcium channel blockers was recorded from each patient’s medication profile. Lower urinary tract symptoms and the severity of incontinence were measured using standardized questionnaires including the International Consultation on Incontinence Questionnaire. The type of incontinence was determined clinically by a physician specialized in incontinence. Co-morbidities were ascertained by self-report. Logistic regression analyses were used to detect factors associated with medication use, as well as relationships between specific medication classes and the type and severity of urinary symptoms.

**Results:**

The prevalence of medications potentially contributing to lower urinary tract symptoms was 60.5%. Calcium channel blockers (21.8%), benzodiazepines (17.4%), other centrally active agents (16.4%), ACE inhibitors (14.4%) and estrogens (12.8%) were most frequently consumed. Only polypharmacy (OR = 4.9, 95% CI = 3.1-7.9), was associated with medication use contributing to incontinence in analyses adjusted for age, sex, and multimorbidity. No associations were detected between specific medication classes and the type or severity of urinary symptoms in this cohort.

**Conclusion:**

The prevalence of use of medications potentially causing urinary symptoms is high among incontinent older adults. More research is needed to determine whether de-prescribing these medications results in improved urinary symptoms.

## Background

Lower urinary tract symptoms such as urinary frequency, urgency, nocturia, urinary incontinence and voiding difficulties are highly prevalent and bothersome, impair quality of life and are associated with falls in the elderly [1-4]. The etiology of lower urinary tract symptoms may be singular or multifactorial, with potential contributors encompassing lifestyle habits, underlying bladder or pelvic floor dysfunction, associated co-morbidities and/or functional factors [5,6]. Many commonly used medications have been proposed to inadvertently interfere with bladder and/or sphincter function, or indirectly impair the ability to toilet [6,7]. The use of oral estrogens, alpha-blocking agents, sedative-hypnotics, antidepressants, antipsychotics, ACE inhibitors, loop diuretics, non-steroidal anti-inflammatory agents (NSAIDS) and calcium channel blockers have all been implicated to some degree in the onset or exacerbation of urinary symptoms [8-23]. Stress urinary incontinence has been associated with the use of alpha-blocking agents, which reduce urethral sphincter tone, and with consumption of ACE inhibitors in patients who experience treatment-related cough [24,25]. Loop diuretics promote polyuria and can lead to urinary urgency and frequency, while NSAIDS and other medications known to cause edema may exacerbate nocturia due to nocturnal fluid redistribution. Centrally active medication potentially exert direct effects on the lower urinary tract system by causing relaxation of striated pelvic floor muscles and interfering with afferent sensory messages from the bladder, or even indirectly through their effect on mobility and toileting ability. Calcium channel blockers have been associated with bladder relaxation and impaired emptying, while the mechanism of action of estrogen replacement therapy remains unknown. Acetylcholinesterase inhibitors can precipitate *de novo* urgency incontinence via enhanced cholinergic activity in the bladder.

Elderly patients who seek care for incontinence have a higher likelihood of multiple morbidity, functional impairment and increased susceptibility to the adverse effects of medications [6]. The prevalence of use of medications potentially contributing to urinary symptoms in older adults seeking care for incontinence remains unknown. A greater understanding of the frequency of use of specific classes of medications among these individuals could help target future research studies and clinical care approaches for reducing medication-risk in patients with urinary symptoms.

The purpose of the present report is to describe the prevalence of use of medications potentially causing or exacerbating lower urinary tract symptoms in a clinical cohort of older men and women presenting to outpatient incontinence clinics for an evaluation of urinary incontinence. Secondary objectives were to determine which factors were associated with the use of these medications, and to investigate whether associations between specific medication classes and the type or severity of urinary symptoms could be detected.

## Methods

### Study design, setting and sample

A cross sectional study of incontinent men and women aged 60 years and older was conducted in Quebec, Canada. Participants were recruited from consecutive new patients presenting to three outpatient urology clinics and three geriatric outpatient incontinence clinics in the Montreal and Sherbrooke areas of Quebec, Canada. The method for recruiting patients has been described previously [26]. Briefly, patients were included in the cohort if they reported a weekly average of one or more episodes of involuntary urine loss during the preceding 3 months. Patients with known dementia (a screening Mini-Mental State Exam score ≤ 24) were excluded from the cohort in order to avoid unreliable self-reported measures. Ten patients with missing data or incomplete medication information were excluded from the analyses. All protocols and procedures were approved by the Institutional Ethics Review Boards of the Institut universitaire de gériatrie de Montréal and the Institut universitaire de gériatrie de Sherbrooke. Informed consent was obtained from each participant prior to data collection.

### Data collection and measurement

A research assistant recorded medication use from bottle labels (or dosette boxes) for prescription drugs and over-the-counter products at the time of the initial clinic visit. Adherence was determined by asking patients if they were currently consuming each medication as prescribed. The name of each prescribed medication, the frequency, daily dose, and treatment duration were recorded. Medications were coded according to the Anatomical Therapeutic Classification [27] for users and non-users of oral estrogens, alpha-blocking agents (antihypertensive and prostate medications), benzodiazepines, antidepressants, antipsychotics, ACE inhibitors, loop diuretics, NSAIDs, lithium, narcotics and calcium channel blockers. Medications known to cause pedal edema such as other GABAergic analgesics (gabapentin, neurontin) and the thiazolinedione hypoglycemic agents were also recorded. Local estrogen formulations were not considered in this analysis.

Storage lower urinary tract symptoms were queried by self-report in accordance with definitions from the International Continence Society [28]. Increased daytime urinary frequency was defined as passing urine 8 or more times per day. Participants reporting the need to wake up at night more than once to urinate were defined as having nocturia. Participants who reported a feeling of urgency and having to rush to the toilet before passing urine (occasionally, most of the time, or all of the time) were designated as having urinary urgency. The International Consultation on Incontinence Questionnaire-ICIQ-UI-SF was used to determine the presence and severity of urinary incontinence [29]. The severity score for each patient was calculated as the sum of the first 3 items (frequency, amount and impact), ranging from 0 to 21 points. Three severity categories were created: mild (0–5), moderate (6–12), and severe (13–21). The type of incontinence was diagnosed by the treating physician after performing a standard assessment including history, physical examination, stress test, bladder ultrasound for post-void residual urine volume, and urine analysis. A diagnosis of stress, urgency, mixed, urgency with impaired bladder emptying, or functional incontinence was relayed to the research team. Urodynamic tests were not performed unless ordered by the treating physician. Voiding symptoms were not systematically recorded and are not considered in this analysis. Comorbidities and other socio-demographic variables were self-reported by the participant to the research assistant. Comorbidities were based on previous medical diagnoses. Heart disease, stroke and peripheral vascular disease were categorized together as cardiovascular diseases. Musculoskeletal disorders included arthritis, osteoporosis and gout.

### Statistical analyses

Descriptive statistics were used to characterize the participants and to determine the prevalence of use of each medication type. Data were summarized by percentages for categorical variables, and means (±standard deviations) for continuous data. To investigate factors associated with the use of medications contributing to lower urinary tract symptoms, logistic regression analyses were conducted, with medication use inserted in the model as the dependent variable. Age (continuous), sex (binary), individual comorbid conditions (binary), multimorbidity (≥ 3 conditions)^29^ and the presence of polypharmacy (≥ 5 medications)^30^ were regressed against the use of each medication class. Medications belonging to classes presumed to contribute to urinary symptoms were excluded from the polypharmacy variable. To determine whether associations could be detected between the type or severity of lower urinary tract symptoms and the use of drugs from specific medication classes, univariate and multivariate analyses were conducted with the independent variable coded as user versus non user of the targeted medications potentially causing or exacerbating lower urinary tract symptoms (e.g. oral estrogens, alpha-blocking agents, sedative-hypnotics, antidepressants, antipsychotics, ACE inhibitors, loop diuretics, NSAIDS, calcium channel blockers, and other GABAergic analgesics). The dependent variable was either the presence or absence of each urinary symptom (urgency versus no urgency, nocturia versus nocturia); the type of incontinence (e.g. stress versus other types, urge versus other types); or the severity of incontinence (mild versus moderate or severe). First we regressed “any” medication class as the independent variable, then subgroup analyses were conducted using each distinct medication class separately. Age, sex, polypharmacy and other co-morbidities were included in multivariate analyses if they were independently associated with the outcome at p < 0.05. Results are presented as odds ratio (OR) and 95% confidence intervals (95% CI). All statistical analyses were conducted using SPSS.

## Results

Three hundred and ninety men and women consented to enroll in the study over a four year time period, representing approximately forty percent of all new patients. The most common reasons for refusing to participate were dementia, institutionalization, disinterest in participating in a research study, and competing acute illness. Data on eligible patients who refused to participate were not available for comparison. Only 1% of interested individuals met exclusion criteria.

Table 1 shows the characteristics of the participants and the distribution of urinary symptoms and types of incontinence. The mean age was 72.4 ± 6.4 years (range 60–90), the mean number of co-morbid conditions was 5.0 ± 2.5, and patients consumed on average 6.2 ± 3.8 different medications. The most frequent co-morbid conditions included musculoskeletal disorders (self-reported arthritis symptoms), dyslipidemia and depression. All patients were incontinent; the majority (93.1%) reported moderate to severe symptoms. Forty-five percent of patients were diagnosed with urgency incontinence as the primary diagnosis, explaining the high prevalence of other overactive bladder symptoms such as urinary frequency (78.7%), urgency (91.8%) and nocturia (83.6%) in the cohort.

**Table 1:** Patient characteristics and distribution of urinary symptoms (n = 390)

**Characteristics**	**Number (%)**
Age in years (Mean ± SD)	72.4 ± 6.4
60-69	150 (38.5%)
70-79	175 (44.8%)
80+	65 (16.7%)
Female	355 (91.0%)
Co-morbid conditions (≥ 3)	315 (80.7%)
Hypertension	118 (30.3%)
Dyslipidemia	166 (42.6%)
Diabetes	93 (23.8%)
Cardiovascular disease	133 (34.1%)
Depression	142 (36.4%)
Respiratory disease	65 (16.7%)
Renal failure	20 (5.1%)
Musculoskeletal disorders	385 (98.7%)
Polypharmacy (≥ 5 medications)	244 (62.6%)
Type of incontinence	
Urgency	175 (45.0%)
Stress	56 (14.4%)
Mixed	98 (25.0%)
Urgency with impaired emptying	16 (4.1%)
Functional	45 (11.5%)
Severity of incontinence	
Mild	27 (6.9%)
Moderate	194 (49.7%)
Severe	169 (43.4%)
Other lower urinary tract symptoms	
Urinary frequency	307 (78.7%)
Urinary urgency	358 (91.8%)
Nocturia	326 (83.6%)

The prevalence of use of medications potentially contributing to urinary symptoms was 60.5% (n = 236), with a mean of one incriminating drug per patient (Table 2). The top five medication classes were calcium channel blockers (21.8%), benzodiazepines (17.4%), other centrally active agents including antipsychotics, antidepressants and narcotics (16.4%), ACE inhibitors (14.4%) and oral estrogens (12.8%). Use of loop diuretics and alpha-blocking antihypertensive agents was rare.

**Table 2:** Prevalence of use of medications potentially contributing to urinary symptoms (n = 390)

**Medication class**	**Prevalence n (%)**
Any medication class	236 (60.5%)
Calcium channel blockers	85 (21.8%)
Benzodiazepines	68 (17.4%)
Other centrally active agents*	65 (16.7%)
ACE inhibitors	56 (14.4%)
Oral estrogens	50 (12.8%)
Oral estrogen + progesterone	12 (3.1%)
NSAIDS	38 (9.7%)
GABAergic analgesics	11 (2.8%)
Loop diuretics	7 (1.8%)
Thiazolinedione hypoglycemic agents	3 (0.8%)
Alpha blocking antihypertensives	1 (0.3%)

*Antidepressants, antipsychotics and narcotics were combined.

Polypharmacy was the only factor associated with the use of medications potentially contributing to incontinence in multivariate analysis (Table 3). Individuals consuming 5 or more drugs were almost 5 times more likely to be taking a medication contributing to urinary symptoms, when adjusting for age, sex and comorbidity (OR = 4.9, 95% CI = 3.1-7.9). Multimorbidity, cardiovascular disease, hypertension, and dyslipidemia were associated with medication use in univariate models but did not remain significant in adjusted models. No associations were found between age or sex and the use of medications potentially contributing to urinary symptoms.

**Table 3:** Factors associated with the use of medications potentially causing urinary symptoms

**Predictor**	**Crude odds ratio**	Adjusted odds ratio*
	**(95% CI)**	**(95% CI)**
Age	1.0 (1.0 - 1.1)	1.0 (1.0 - 1.1)
Sex	1.5 (0.7 - 3.0)	1.1 (0.5 - 2.3)
Polypharmacy (≥ 5 drugs)	5.3 (3.4 – 8.2)	4.9 (3.1 – 7.9)
Multimorbidity (≥ 3)	2.0 (1.2 - 3.3)	1.2 (0.6 – 2.03)
Depression	1.1 (0.7 - 1.67)	0.7 (0.4 - 1.2)
Cardiovascular disease	2.0 (1.3 - 3.0)	1.4 (0.8 - 2.3)
Hypertension	2.2 (1.4 - 3.5)	1.5 (0.9 - 2.7)
Respiratory disease	1.7 (1.0 - 3.1)	1.4 (0.7 - 2.9)
Gastrointestinal disorders	1.5 (1.0 - 2.2)	1.1 (0.6 - 1.9)
Dyslipidemia	1.6 (1.1 - 2.4)	0.9 (0.5 - 1.5)
Renal failure	1.6 (0.6 - 4.1)	0.7 (0.2 - 2.1)
Musculoskeletal disorders	1.3 (1.0 - 1.6)	0.9 (0.6 - 1.2)
Diabetes	1.4 (0.9 - 2.3)	0.9 (0.5 - 1.7)

*Adjusted for age, sex, polypharmacy and multimorbidity.

Regression analyses failed to detect significant associations between urinary frequency, urgency or nocturia with any medication class. Nor was any association found between the class of medication and the type or severity of incontinence in univariate or multivariate models adjusted for age, sex, polypharmacy and multiple morbidity (data not shown).

## Discussion

The prevalence of use of medications potentially contributing to urinary symptoms was high (60.5%) among 390 new patients presenting for evaluation to a geriatric incontinence clinic. The most common medication classes were calcium channel blockers, benzodiazepines, other centrally active agents, ACE inhibitors and oral estrogens. Loop diuretics and alpha-blocking agents were less frequently prescribed. Patients with polypharmacy were five times more likely to consume potentially deleterious medications, regardless of age, sex and multimorbidity. No specific disease state was associated with medication use when polypharmacy was accounted for. Our analyses were unable to detect significant associations between specific medication classes and the type or severity of lower urinary tract symptoms.

The lack of association found in the current study between specific medication classes thought to precipitate symptoms and the type or severity of symptoms mirrors the negative results reported from other large epidemiological studies that examined the relationship between current medication use and urinary incontinence, such as the Boston Area Community Health Survey and the Health, Aging and Body Composition Study [10,21]. One explanation is the cross-sectional nature many of these analyses. Studies that do not account for the timing of administration of a new drug in relation to the appearance of urinary symptoms are limited in their ability to establish causality. Criteria for causality require that the cause precede the effect [30,31]. To meet this criterion, neither incontinence nor the use of a medication class thought to contribute to urinary symptoms should be present at the onset of the study in the population of interest. Observational studies that aim to investigate the effect of specific medication classes on the occurrence and type of urinary symptoms in subgroups of healthy and frail individuals should employ a new-user cohort of individuals without previous medication use or urinary symptoms, rigorous ascertainment of incident incontinence, and sensitive markers of frailty [32]. The strength of the association and the specificity of different treatment-emergent harmful effects in elderly individuals may vary according to whether individuals are healthy, frail or pre-frail, and whether more than one urinary symptom develops. The risk of adverse outcomes may therefore depend on the presence of multiple risk factors and these should be accounted for in the analysis.

Despite this research complexity, a growing body of evidence supports clinicians in their efforts to pursue medication modification as a risk reduction strategy for patients experiencing urinary symptoms. Data exist on post-menopausal hormone therapy examined in the context of two large randomized placebo controlled trials, with urinary symptoms as a post-hoc endpoint [8,9]. Findings indicated a two-fold increased risk of *de novo* stress and mixed incontinence among new users of estrogen replacement therapy [8], a 30% increase in the onset of urgency incontinence [8] and a significant exacerbation in the amount and frequency of stress and urgency incontinence among women with pre-existing symptoms [8,9]. In a case control study, 20/49 women taking alpha-blocking antihyperstensives experienced incontinence compared to 8/49 controls (40.8 vs. 16.3%), although the relationship no longer persisted when patients on loop diuretics were excluded from the analysis [12]. Discontinuation of alpha-blocking agents in 18 women with incontinence resulted in 13 (72%) reporting total or almost complete resolution of their urinary symptoms [12]. Clinical data from a series of 172 elderly hypertensive and heart failure patients found that the use of loop but not thiazide diuretics was significantly associated with increased urinary frequency, a relationship that persisted when the age of the patient and the use of other cardiovascular diseases were accounted for [16]. New use of calcium channels blockers among 38 men aged 47–89 years old was found to significantly exacerbate voiding symptoms, even when adjusting for other medication use and conditions that could contribute to lower urinary tract symptoms [23]. A number of case reports describe cough-induced stress incontinence upon initiation of an ACE inhibitor, which remits upon discontinuation, and one case series reported a 10% incidence of severe ACE-inhibitor-induced stress incontinence in diabetic post-menopausal women [17,24,25].

The prevalence of use of medications potentially affecting lower urinary tract symptoms may be underestimated in the current study due to selection bias. Patients with dementia, residents of nursing homes, and acutely ill patients did not participate, and may reasonably have a higher risk of taking acetylcholinesterase inhibitors or consuming multiple medications. Furthermore, the cohort comprised a select sample of patients who were referred to a specialist clinic, and only 40% of eligible patients consented to provide data. Women were over-represented. Many patients are referred to subspecialty clinics only when their symptoms are refractory to first-line management, so medications contributing to urinary symptoms already may have been discontinued. Patients successfully treated by a primary care provider by stopping a medication (e.g., a diuretic), would not be referred to the incontinence clinic and would not be included in the current sample. Multimorbidity was not a significant predictor when polypharmacy was accounted for, and this could partially be explained by collinearity between the two variables, or by virtue of polypharmacy acting as a proxy of disease severity. Although an attempt was made to investigate the association between medication usage and the type of incontinence, the etiology of incontinence is often multifactorial in older persons, so misclassification or lack of specificity of the type of incontinence may have contributed to the null effect of these analyses.

## Conclusion

The prevalence of use of medications potentially causing or exacerbating lower urinary tract symptoms was high (60.5%) in this clinical cohort of new patients presenting for a geriatric evaluation of urinary incontinence. More research is needed to determine whether de-prescribing these medications results in improved urinary symptoms.

## Competing interests

The authors have no financial conflicts of interest to disclose with respect to this work. Cara Tannenbaum declares having received honoraria from Pfizer, Allergen and Ferring Pharmaceuticals during the past three years. Le Mai Tu declares having consulted for Pfizer, Astellas, Watson, Medtronic and Allergen. Although both Cara Tannenbaum and Le Mai Tu have served as consultants or speakers at symposium for these companies related to the pharmacologic treatment of incontinence in the elderly with bladder relaxant medication, the authors do not feel that they were influenced in any way in the reporting of the results from the current clinical cohort study (which was not funded by industry).

## Authors’ contributions

Study concept and design: CT and MK; Acquisition of subjects and/or data: CT and LMT; Analysis and interpretation of data: MK and CT; Preparation of manuscript: MK, CT and LMT. All authors read and approved the final manuscript.

## Pre-publication history

The pre-publication history for this paper can be accessed here:

http://www.biomedcentral.com/1471-2318/13/57/prepub

## Meeting attended and presented

American Geriatric Society conference 2012 Seattle, WA, USA.
